# Lumbosacral Erector Spinae Plane Block Versus Psoas Muscle Compartment with Sciatic Nerve Block for Anesthesia for Unilateral Lower Limb Operations in Critically Ill Patients: A Randomized Open-Label Study

**DOI:** 10.5812/aapm-165030

**Published:** 2025-09-07

**Authors:** Mohamed R. Elbasyouny, Mohsen M. Eissa, Mahmoud R. Zomra, Mohamed Ali Mahmoud, Mohammed Said ElSharkawy, Ashraf E. Abdalla

**Affiliations:** 1 Anesthesiology, Surgical Intensive Care and Pain Management Department, Faculty of Medicine, Al-Azhar University, Cairo, Egypt; 2 Anesthesia, Intensive Care and Pain Management Department, Faculty of Medicine, Al-Azhar University, Cairo, Egypt; 3 Anesthesiology, Surgical Intensive Care and Pain Management Department, Faculty of Medicine, Tanta University, Tanta, Egypt

**Keywords:** Anesthesia, Critically Ill, Erector Spinae Plane Block, Lower Limb Surgery, Psoas Compartment Block, Sciatic Nerve Block

## Abstract

**Background:**

Regional anesthesia techniques have gained popularity for lower limb (LL) surgeries. The psoas compartment block (PCB)-sciatic nerve block (SNB) combination effectively anesthetizes the entire lower extremities while providing greater hemodynamic stability. The combined lumbosacral erector spinae plane block (LS-ESPB) has shown promise in providing effective analgesia for various surgical procedures by targeting both the lumbar and sacral regions.

**Objectives:**

This investigation compared the outcomes of the LS-ESPB and the PCB combined with the SNB for unilateral LL anesthesia.

**Methods:**

This randomized open-label study involved 130 critically ill patients, of both sexes, aged 18 - 65 years, undergoing unilateral LL operations. Participants were randomized equally into two groups. Group A received ultrasound (US)-guided lumbar erector peripheral nerve block (LESPB, 20 mL) and sacral erector peripheral nerve block (SESPB, 40 mL), and group B received US-guided PCB (20 mL) combined with SNB (20 mL). Both groups received blocks of 0.25% bupivacaine.

**Results:**

The incidence of success was significantly higher in group A compared to group B (90.77% vs. 76.92%, P = 0.032). Intraoperative heart rate (HR) and mean arterial blood pressure (at 30 and 45 min), pain scores (at 6h), and total morphine consumption within the first 24 hours were significantly lower in group A compared to group B (P < 0.05). The time to the first request for rescue analgesia was significantly prolonged in group A compared to group B (P < 0.001). Side effects were similar within the studied groups. Patient satisfaction was considerably higher in group A compared to group B (P = 0.020)

**Conclusions:**

The LS-ESPB is more effective than the PCB-SNB combination for unilateral LL surgeries, offering a higher incidence of success, more stable hemodynamics, better analgesia, and higher patient satisfaction, with similar side effects.

## 1. Background

Anesthesia for lower limb (LL) surgeries in critically ill patients requires careful consideration due to the increased risk of complications associated with general and neuraxial techniques, such as hemodynamic instability, respiratory depression, and infection (1). General anesthesia in critically ill patients presents considerable risks due to their compromised health, rendering them more vulnerable to complications such as respiratory failure, circulatory instability, renal and hepatic dysfunction, and neurological issues, including delirium (2).

Neuraxial anesthesia can be adequate for pain management and anesthesia in surgical procedures, but its use in critically ill patients carries significant risks and potential complications. Key complications include an increased risk of infection, hemodynamic instability, neurological issues, coagulation problems, respiratory depression, and urinary retention (3).

Currently, there is growing interest in peripheral nerve blocks (PNBs) as a safer alternative for anesthesia and pain management in LL surgeries (4). The PNBs provide substantial benefits by improving safety, enhancing efficacy, and accelerating recovery outcomes. The PNBs reduce the risk of respiratory and cardiac complications (5). The psoas compartment block (PCB) is a regional anesthesia approach targeting the main branches of the lumbar plexus (6). The PCB-sciatic nerve block (SNB) combination effectively anesthetizes the entire lower extremities while providing greater hemodynamic stability (7).

The erector spinae plane block (ESPB) allows for the distribution of local anesthesia (LA) cranially and caudally, encompassing up to nine dermatomes (8) and then reaching the paravertebral space and rami of the spinal nerves (both the ventral and dorsal) (9). Sacral (SESPB) and lumbar erector spinae plane block (LESPB) are employed in various surgical approaches, such as LL surgeries (10). The combined lumbosacral erector spinae plane block (LS-ESPB) has shown promise in providing effective analgesia for various surgical procedures by targeting both the lumbar and sacral regions. Marrone et al. (11) demonstrated that the LS-ESP block significantly diminished postoperative pain scores and the need for rescue analgesia in hip fracture surgery.

We hypothesized that LS-ESPB is a more effective analgesic approach than PCB in unilateral LL surgeries for critically ill patients. Upon reviewing existing literature, we found a deficiency in studies directly comparing the effectiveness of LS-ESPB and PCB with SNB in unilateral LL operations for critically ill patients.

## 2. Objectives

This study compared LS-ESPB and PCB combined with SNB outcomes in these specific surgical cases.

## 3. Methods

In this randomized, open-label study, we compared the efficacy of the LS-ESPB and PCB combined with SNB in critically ill patients who were admitted to the ICU and undergoing unilateral LL surgery. This randomized open-label research was conducted on 130 critically ill patients, comprising both sexes, aged 18 - 65 years, with physical status categorized as III-IV by the American Society of Anesthesiologists (ASA), undergoing unilateral LL operations. The research was carried out from August 2024 to March 2025, after receiving approval from the Ethics Committee of Tanta University Hospitals (approval code: 36264PR761/7/24) and registering on ClinicalTrials.gov (ID: NCT06580704), (date of registration: 30/8/2024). This study was conducted in accordance with the Declaration of Helsinki. Informed written consent was obtained from all participants prior to enrollment. Participants were excluded if they had any allergies to LA, bleeding disorders, coagulopathy, were on anticoagulant therapy, had pre-existing psychological, neurological, or spinal cord conditions, prior back surgeries, severe cardiac or renal diseases, or were on chronic analgesic medications. Additionally, individuals with infections or tumors at the site of the block were also excluded.

Before the surgery, participants underwent thorough medical history assessments, clinical examinations, and laboratory testing. They were also educated on how to use the Visual Analog Scale (VAS) to rate their pain levels, ensuring accurate pain reporting throughout the study. All patients received 3 mg of midazolam for sedation.

### 3.1. Randomization

For randomization, a computer-generated random allocation process (https://www.randomizer.org/) was employed, and patient codes were placed in sealed, opaque envelopes to preserve blinding. Participants were randomly assigned to one of two equal groups: Group A, which received ultrasound (US)-guided ESPB, or group B, which received US-guided PCB combined with SNB. An open-label design was chosen due to the different block techniques used in each group.

Upon arrival at the operating room, an intravenous (IV) line was established, and routine monitoring was initiated, including pulse oximetry, non-invasive blood pressure, electrocardiogram (ECG), and temperature probes. Additional monitoring was applied as needed based on the clinical condition of the patient. The nerve blocks were performed using a US machine (Philips CX50, Amsterdam, Netherlands) equipped with a 6 - 12 MHz linear probe, ensuring precise and sterile conditions. Before the blocks, the skin entry point was infiltrated with 1% lidocaine. A negative aspiration test was performed to confirm the absence of intravascular placement.

### 3.2. The Lumbosacral Erector Spinae Plane Block Procedure

For the LESPB, the procedure was conducted at the L3 - L4 vertebral level on the surgical side, with the patient positioned laterally. A longitudinal alignment of the US probe was maintained in the sagittal plane along the mid-vertebral line. The probe was moved laterally by 3.5 - 4 cm to visualize the ES muscle next to the transverse process (TP). A 22-gauge needle was inserted with its bevel oriented cephalocaudally, and hydro-dissection was performed with 0.5 - 1 mL of saline, ensuring accurate needle placement in the fascial plane near the ES muscle. The final position was confirmed when the hyperechoic shadow of the TP was observed. After ensuring proper needle placement, 20 mL of 0.25% bupivacaine was administered. For the SESPB, the needle was introduced in a cranial-to-caudal direction using an in-plane technique, targeting the S4 crest. At this location, 20 mL of 0.25% bupivacaine was administered.

This procedure was subsequently repeated at the S2 median sacral crest, where an additional 20 ml of bupivacaine was injected. In total, 40 mL of LA was administered at the sacral level. The LA was carefully deposited in the fascial plane located between the ES muscles and the sacral crests (12).

### 3.3. Psoas Compartment Block Procedure

For the PCB procedure, the US probe was positioned at the L3 - L4 level in the transverse plane to visualize the vertebral body, articular processes, psoas, and other relevant muscles. A 22G needle was inserted laterally under US guidance, confirming needle placement via quadriceps muscle contraction. We used neurostimulation to assess quadriceps contraction in the PCB group. The motor response in the quadriceps muscle was observed upon the injection of the local anesthetic. If no muscle contraction was detected, the block was considered successful. Incremental doses of 20 mL of 0.25% bupivacaine were administered, with aspiration after each injection to confirm the absence of intravascular injection.

For the SNB, patients were positioned supinely with the affected knee flexed to approximately 30 degrees to facilitate access to the target nerve. The procedure was performed under US guidance, enabling real-time visualization of the sciatic nerve in a short-axis view. The nerve was traced from its origin at the popliteal fossa along the posterior thigh, and it was located posterior-laterally to the popliteal artery at the knee. At the mid-thigh region, the nerve appeared as a rounded, echogenic structure on the US image. The block was administered at the site where the sciatic nerve bifurcates into the tibial and common peroneal nerves. A 22-gauge needle was introduced from lateral to medial, positioned anterior to the biceps femoris tendon, and aligned parallel to the US beam. This ensured the needle trajectory was optimally placed for accurate needle tip positioning. The needle tip was advanced to target both the anterior and posterior surfaces of the sciatic nerve in the anterior-posterior plane, ensuring that the needle was centered in the mediolateral plane. Once the needle was correctly positioned, a total of 20 mL of 0.25% bupivacaine was injected around the nerve to achieve the desired anesthetic effect.

### 3.4. Assessment of Block Success and Dermatomal Coverage

Sensory block was assessed 30 minutes post-injection using pinprick testing across T12-S4 dermatomes, focusing on all LL regions. The LESPB consistently covered T12-L4, while SESPB showed variable spread over S2 - S4, mainly in the posterior thigh and gluteal areas. Complete sciatic territory coverage, especially below the knee, was inconsistent in LS-ESPB. In contrast, PCB+SNB provided uniform, dense anesthesia across both lumbar and sciatic distributions. Surgery commenced after confirming block efficacy. A > 20% rise in heart rate (HR) or mean arterial pressure (MAP) during stimulation indicated inadequate analgesia, managed with fentanyl (0.5 μg/kg). Repeat dosing or persistent pain prompted conversion to GA. A block was considered successful if three criteria were met: Adequate sensory loss (T12-S4), stable HR/MAP, and no fentanyl dose > 0.5 μg/kg or repeated dosing. Failures were excluded from the incidence of success analysis.

To ensure consistent patient stability throughout the surgical procedure, MAP and HR were carefully monitored at baseline and subsequently at regular 15-minute intervals throughout the duration of the surgery. This monitoring was conducted to assess any fluctuations and promptly address any potential complications that could arise during the procedure. In the postoperative phase, a standardized analgesic protocol was strictly adhered to in order to ensure optimal pain management for all patients involved in the study. Specifically, all patients were administered IV paracetamol at a dosage of 15 mg/kg every 8 hours, which was part of the routine analgesic regimen designed to provide continuous pain relief.

In cases where the VAS pain score exceeded 3, rescue analgesia in the form of a 3 mg morphine bolus was administered. If pain persisted and the VAS score remained elevated, the morphine dose could be repeated every 30 minutes, with the goal of reducing the pain score below 4. Pain assessments were conducted at multiple points throughout the postoperative period, ensuring that pain levels were closely monitored. These assessments took place in the post-anesthesia care unit (PACU) immediately following surgery and were also performed at 2, 4, 8, 12, and 24 hours postoperatively to evaluate the ongoing effectiveness of the analgesic regimen.

In addition to pain management, a thorough and vigilant monitoring system was put in place to detect any adverse side effects or complications that might arise following surgery. Specifically, the occurrence of hypotension was defined as a MAP of less than 65 mmHg or a decrease of 20% or more from baseline values. In the event of hypotension, the condition was promptly managed with a dose of 10 mg ephedrine to restore stable blood pressure. Bradycardia, characterized by a HR dropping below 50 beats per minute, was managed using IV atropine at a dosage of 0.02 mg/kg to ensure appropriate HR levels. Respiratory depression, identified by a SpO_2_ level falling below 95%, was addressed by providing oxygen supplementation to ensure the patient’s oxygen levels remained adequate. Postoperative nausea and vomiting (PONV), a common side effect of anesthesia, was treated with IV ondansetron at a dosage of 0.1 mg/kg to alleviate symptoms. Additionally, any other complications associated with the nerve blocks or other aspects of the surgical procedure were closely monitored and managed promptly by the clinical team.

Patient satisfaction was assessed using a 5-point Likert scale, where patients rated their satisfaction as follows: 1 = extremely unsatisfied, 2 = unsatisfied, 3 = neutral, 4 = satisfied, and 5 = extremely satisfied (13). The patient satisfaction survey was conducted 24 hours after surgery and evaluated various domains of satisfaction, including pain management (effectiveness of analgesia and need for rescue medication), anesthesia effectiveness (success of the block and absence of intraoperative pain), side effects (such as PONV), overall comfort during the surgery, and recovery experience in the PACU. All side effects, including bradycardia, hypotension, PONV, hematoma, local anesthetic systemic toxicity (LAST), or any other complications, were carefully recorded. The primary outcome of the study was the incidence of success of the blocks, while the secondary outcomes included intraoperative hemodynamic parameters, postoperative pain scores, duration of analgesia, patient satisfaction, and the incidence of adverse events.

### 3.5. Sample Size Calculation

The sample size was determined using the statistical software G*Power 3.1.9.2 (Universitat Kiel, Germany). A pilot study was conducted with ten patients per group, which showed an incidence of success of 90% for the LS-ESPB and 70% for the PCB. To calculate the required sample size, several factors were considered, including a confidence level of 95%, statistical power of 80%, and an equal 1:1 ratio for both groups. Additionally, three extra participants were added to each group to account for any potential dropout, ensuring the robustness of the study. This led to a final target of 65 participants in each group.

### 3.6. Statistical Analysis

Data analysis was carried out using SPSS v27 (IBM^©^, Armonk, NY, USA). To evaluate the normality of the data, both the Shapiro-Wilk test and visual inspection via histograms were utilized. For parametric data, means and standard deviations (SD) were computed, with intergroup comparisons made using an unpaired Student’s *t*-test. In cases of non-parametric data, the median and interquartile range (IQR) were used, and differences between groups were assessed with the Mann-Whitney U test. Qualitative data were expressed as frequencies and percentages, and comparisons were conducted using either the chi-square test or Fisher’s exact test, depending on the data’s nature. We used the intention-to-treat analysis for demographic data, while adhering to a per-protocol approach for the other tables. Statistical significance was set at a P-value of less than 0.05, and all tests followed a two-tailed methodology.

## 4. Results

**Figure 1.**
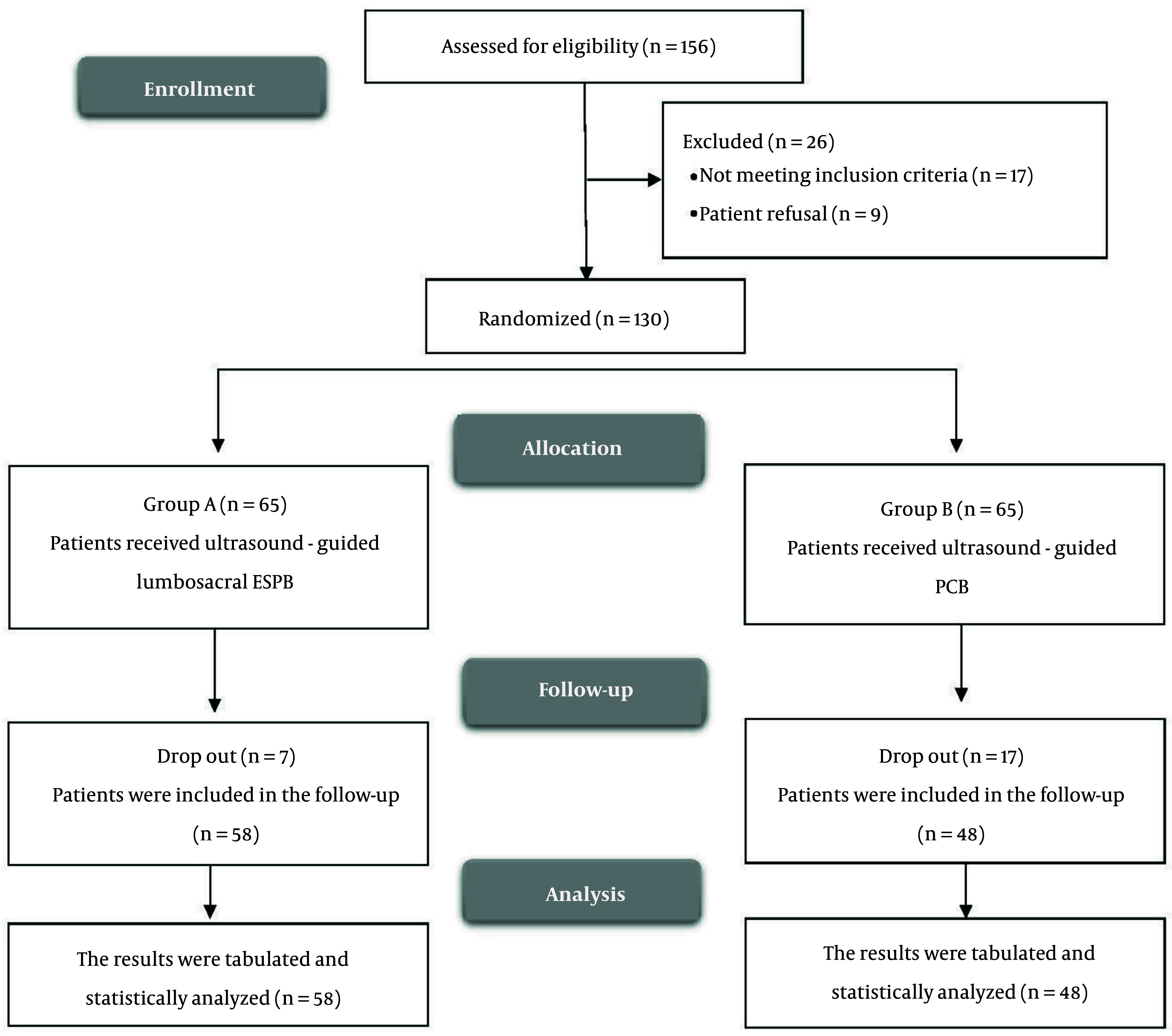
CONSORT flowchart of the enrolled patients

Figure 1 illustrates the enrollment and screening process, wherein a total of 156 cases were initially considered for participation in the study. Of these, 17 patients were excluded due to failure to meet the predetermined inclusion criteria, while an additional 6 patients declined to participate. Consequently, 130 participants were randomized into two groups for subsequent evaluation and analysis in accordance with the intention-to-treat principle. This preserved the original randomization and avoided bias in outcome comparisons, even in the presence of procedural failure (notably elevated in the PCB+SNB group).

Table 1 exhibits the baseline demographic characteristics, type of surgery, and duration of surgery for both groups, revealing no statistically significant differences, thereby confirming the comparability of the groups at baseline.

**Table 1.** Demographic Data, Type of Surgery and Duration of Surgery of the Studied Groups ^a^

Variables	Group A (N = 65)	Group B (N = 65)	P
**Age (y)**	43.78 ± 12.1	41.6 ± 12.12	0.306
**Sex**			0.592
Male	40 (61.54)	37 (56.92)	
Female	25 (38.46)	28 (43.08)	
**Weight (kg)**	74.06 ± 10.62	76.71 ± 8.3	0.116
**Height (cm)**	167.83 ± 7.35	168.71 ± 6.2	0.463
**BMI (kg/m** ^**2**^ **)**	26.4 ± 4.15	26.99 ± 2.93	0.353
**ASA physical status**			0.458
III	45 (69.23)	41 (63.08)	
IV	20 (30.77)	24 (36.92)	
**Type of surgery**			0.815
Knee abscess drainage	8 (12.31)	5 (7.69)	
Femoral-popliteal bypass	10 (15.38)	13 (20)	
Internal fixation of fracture acetabulum	12 (18.46)	10 (15.38)	
Closed reduction and internal fixation of fracture femur	15 (23.08)	18 (27.69)	
Amputation	20 (30.77)	19 (29.23)	
**Duration of surgery (min)**	62.69 ± 12.56	59.85 ± 8.61	0.134

Abbreviations: BMI, Body Mass Index; ASA, American Society of Anesthesiologists.

^a^ Values are expressed as mean ± standard deviations (SD) or No. (%).

**Figure 2.**
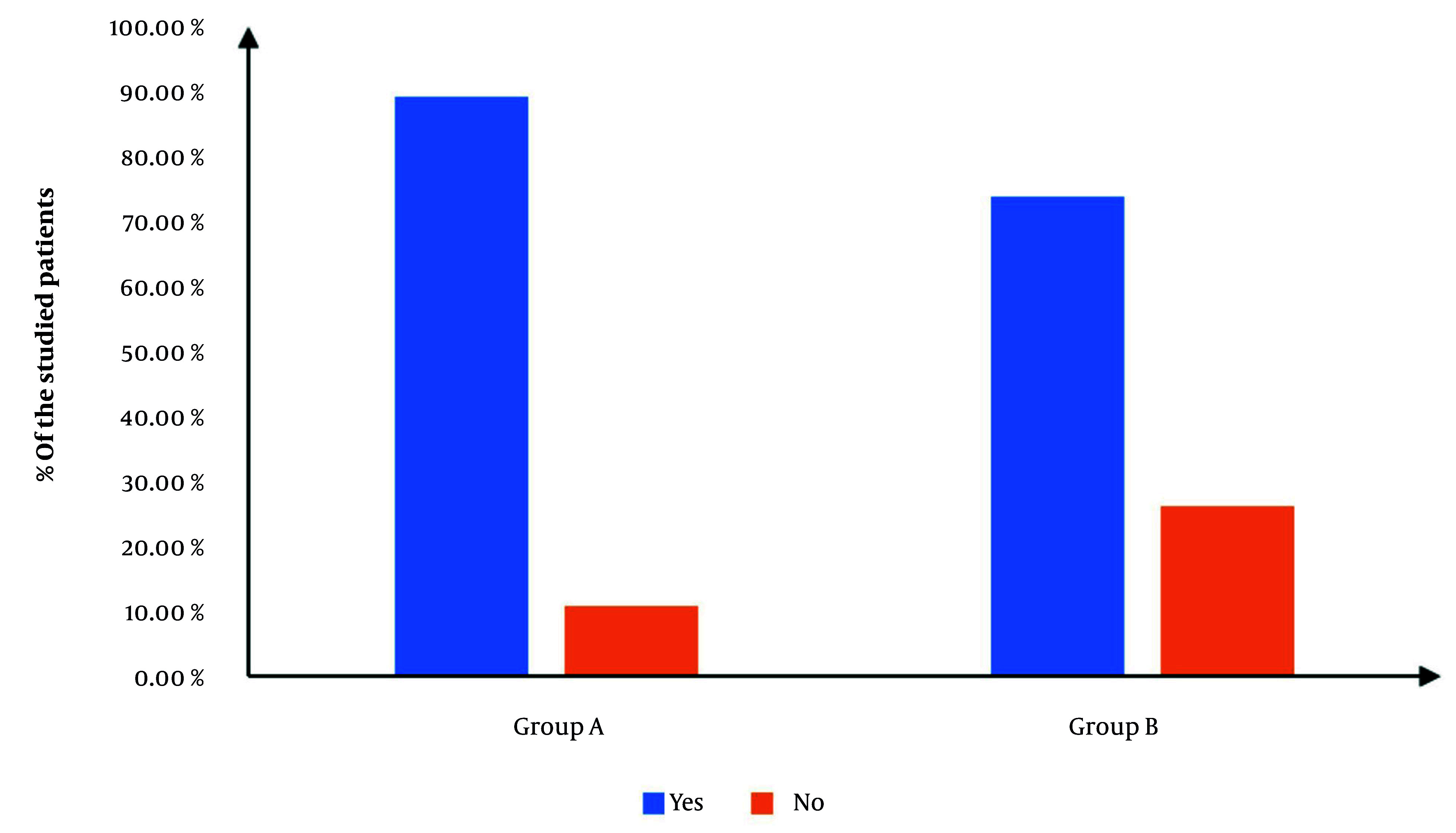
Incidence of success of the studied groups

Figure 2 exhibits the procedural incidence of success, which was significantly elevated in group A in contrast with group B (90.77% vs. 76.92%, P = 0.032), yielding a relative risk (RR) of 0.4 (95% CI: 0.17 to 0.97). Cases in which the procedure failed were excluded from subsequent analysis to maintain the integrity of outcome evaluation.

**Figure 3.**
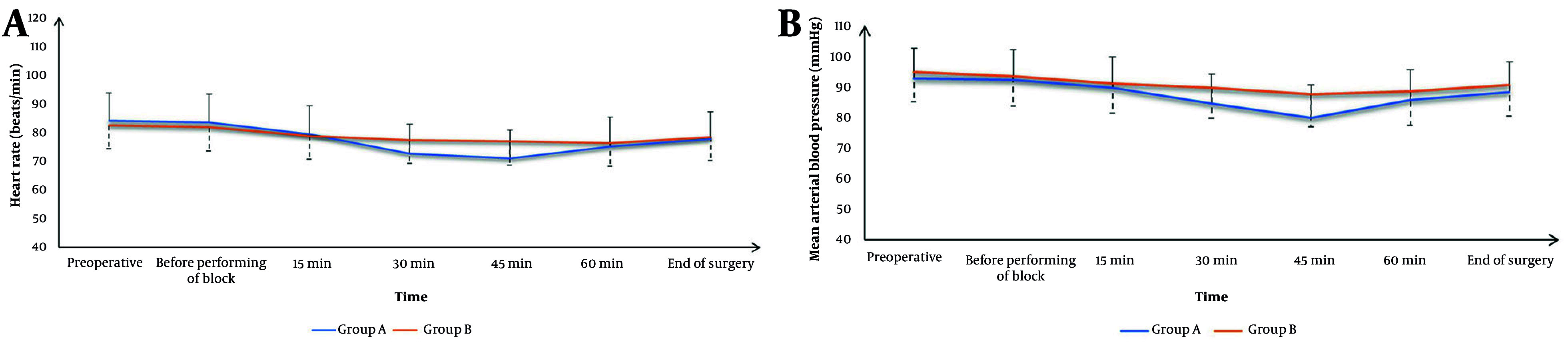
A, heart rate (HR); and B, mean arterial blood pressure changes of the studied groups

Figure 3 demonstrates that HR and MAP were comparable between the two groups at baseline, before block administration, at 15 and 60 minutes intraoperatively, and at the end of surgery. However, at the 30- and 45-minute intraoperative time points, group A exhibited significantly lower HR and MAP values than group B (P < 0.05).

Table 2 reports that the intraoperative fentanyl dose was significantly lower in group A than in group B (P = 0.029). Postoperative pain scores were assessed using the VAS. The scores were statistically similar in both groups in the PACU and at 2, 8, 12, and 24 hours postoperatively. Nevertheless, at the 6- and 8-hour time points, VAS scores in group A were significantly lower compared to those in group B (P < 0.05). Moreover, group A demonstrated a significantly diminished total morphine requirement within the first 24 hours postoperatively (P < 0.05) and a significantly prolonged duration until the first request for rescue analgesia (P < 0.001).

**Table 2.** Analgesic Outcomes of the Studied Groups ^a^

Variables	Group A (N = 58)	Group B (N = 48)	P
**Intraoperative fentanyl dose (kg)**	16.75 ± 17.42	23.72 ± 18.59	0.029
**VAS**			
PACU	1 (0 - 1)	0 (0 - 1)	0.359
2 h	1 (0.25 - 1)	1 (1 - 1)	0.564
4 h	2 (1 - 2)	2 (2 - 3)	0.002
6 h	2 (1 - 2)	2 (2 - 3)	< 0.001
8 h	2 (1 - 4)	3 (2 - 4)	0.134
12 h	2 (2 - 3)	2.5 (2 - 4)	0.652
24 h	3 (2 - 4)	3 (2 - 4)	0.306
**Time to first request of rescue analgesia (h)**	10.76 ± 2.48	7.92 ± 1.65	< 0.001
**Total dose of morphine consumption in the 1st 24 hours (mg)**	4.14 ± 1.47	5.25 ± 1.69	< 0.001

Abbreviations: VAS, Visual Analog Scale; PACU, post-anesthesia care unit.

^a^ Values are expressed as mean ± standard deviations (SD) or median [interquartile rang (IQR)].

Table 3 demonstrates the incidence of adverse effects and patient satisfaction. There were no significant differences between the two groups regarding the occurrence of bradycardia, hypotension, or PONV. Notably, no cases of hematoma or LAST were observed in either group. Patient satisfaction scores were significantly higher in group A compared to group B (P = 0.020).

**Table 3.** Side Effects and Patients’ Satisfaction of the Studied Groups ^a^

Variables	Group A (N = 58)	Group B (N = 48)	P
**Side effects**			
Bradycardia	2 (3.45)	0 (0)	0.492
Hypotension	5 (8.62)	1 (2.08)	0.155
PONV	3 (5.17)	6 (12.5)	0.364
Hematoma	0 (0)	0 (0)	-
LAST	0 (0)	0 (0)	-
**Patients’ satisfaction**			0.020
Extremely satisfied	18 (31.03)	8 (16.67)	
Satisfied	25 (43.1)	13 (27.08)	
Neutral	9 (15.52)	16 (33.33)	
Unsatisfied	4 (6.9)	10 (20.83)	
Extremely dissatisfied	2 (3.45%)	1 (2.08%)	

Abbreviations: PONV, postoperative nausea and vomiting; LAST, local anesthetic systemic toxicity.

^a^ Values are expressed as No. (%).

## 5. Discussion

The analgesic effects of ESPB likely arise from multiple mechanisms, primarily the direct action of LA on sensory nerves within the erector spinae fascial plane (14). Although LA may spread to the paravertebral or epidural spaces, the clinical efficacy is mainly attributed to the blockade of the dorsal and ventral rami, including lateral cutaneous branches and anterior rami (14). Anti-inflammatory effects and systemic absorption may contribute to pain relief, but these are unlikely to explain the rapid, dermatomal onset of analgesia typically observed with ESPB (15, 16).

In contrast, PCB with SNB targets the lumbar plexus and sciatic nerves separately. The PCB anesthetizes the femoral, obturator, and lateral femoral cutaneous nerves by injecting LA within the psoas major muscle (17), while the SNB covers the posterior thigh and most of the lower leg and foot (18). This combination is particularly advantageous in critically ill patients due to its effectiveness in providing dense anesthesia and maintaining hemodynamic stability (19). It has also been shown to diminish opioid use and offer more consistent obturator nerve coverage than a femoral nerve block alone (20).

To enhance sacral coverage in LS-ESPB, we employed a dual injection technique at the S2 and S4 levels, aiming for a more complete blockade of sacral roots and improved diffusion of LA (21). We used 0.25% bupivacaine based on its efficacy in providing sufficient sensory analgesia while minimizing motor blockade, which is essential in critically ill populations. This concentration has proven effective in similar high-risk surgical contexts (22).

The elevated incidence of success of ESPB could be attributed to its relatively more straightforward technique and the broader spread of LA in the fascial plane, potentially covering a larger area of innervation (14). The ESPB affects the fascial plane deep into the ES muscle at the lumbar level. The ESPB is more superficial and more accessible to visualize using US guidance (23) in contrast with the PCB, which requires deeper needle insertion into the psoas muscle compartment (24). Additionally, in SNB, the LA spread is limited to the area surrounding the sciatic nerve itself, which can be restrictive depending on the surgical site (25).

Intraoperative hemodynamics showed significantly lower HR and MAP in the ESPB at 30 and 45 minutes compared to the PCB. Patient satisfaction was significantly higher in the ESPB than in the PCB. This hemodynamic stability could benefit critically ill patients, as it may decrease the risk of cardiovascular complications. Similar hemodynamic advantages of ESPB have been reported by the study conducted by Nagy et al. (26), which evaluated US-guided ESPB efficacy on patient satisfaction and intraoperative hemodynamics. Their findings indicated that US-guided ESPB enhanced perioperative hemodynamic control and stability and improved patient satisfaction.

Abotaleb et al. (27) stated that the US-guided ESPB provided adequate analgesia with more hemodynamic stability than the caudal block in pediatrics undergoing LL surgery. Furthermore, Medhat et al. (28) demonstrated that the LESPB provided elevated patient satisfaction levels in the elderly undergoing hip arthroplasty compared to the control. Moreover, Another study investigated the effectiveness of US-guided ESPB for pain management in lumbar laminoplasties (29) . Their findings indicated that patients receiving ESPB exhibited more stable hemodynamics and greater satisfaction levels compared to those who underwent general anesthesia alone. Additionally, Aksoy et al. (30) illustrated that in elderly high-risk cases undergoing hip replacement surgeries, PCB resulted in significantly elevated MAP values compared to continuous spinal anesthesia (SA).

Postoperative pain control was superior in the ESPB compared to the PCB at 4 and 6 hours postoperatively, with a prolonged latency to the initial demand for rescue analgesia and significantly lower total morphine consumption within the first 24 hours. Consistent with our findings, Marrone et al. (11) reported that incorporating the LESPB into the SESPB significantly enhanced the quality of anesthesia for hip procedures compared to using a combination of PENG and SESPB. This approach proved effective for hip surgery by mitigating the risks associated with neuraxial or general anesthesia, and it provided postoperative analgesia for up to 48 hours without the need for opioids.

Abotaleb et al. (27) reported that in pediatric LL surgeries, US-guided LESPB provided better postoperative pain management compared to the caudal block. Fu et al. (31) agreed with our findings and stated that in lumbar spinal surgeries, the ESPB demonstrated diminished VAS scores and total opioid consumption, as well as an extended duration before the initial need for analgesia compared to the control. Additionally, Zelenty et al. (32) investigated the utility of US-guided ESPB for postoperative pain management in thoracolumbar spinal fusion surgeries. Their findings indicated enhanced pain relief and decreased opioid intake within the initial 24 hours in the ESPB group.

Furthermore, Gani̇dagli et al. (33) showed that in comparison to the femoral-sciatic approach, the PCB-sciatic technique for arthroscopic surgeries provided superior anesthesia and better analgesia. However, Canakci et al. (34) reported that the PCB provides a longer duration before the initial requirement for analgesia and results in lower opioid consumption compared to SA in total knee arthroplasty surgery.

Ilfeld et al. (35) discovered that both continuous posterior lumbar plexus blocks and continuous femoral nerve blocks effectively alleviate pain in adults after hip arthroplasties. In a related study, Marino et al. (36) reported that continuous lumbar plexus and femoral blocks decrease the requirement for opioid analgesics post-surgery. The safety profile of both approaches was similar, with a comparable incidence of side effects (bradycardia, hypotension, PONV, hematoma, or LAST). Fu et al. (31) agreed with our results and demonstrated that ESPB diminished the incidence of PONV compared to the control.

The research is limited by the small sample size, single-center settings, and short-term follow-up (24 hours). Additionally, the open-label design restricted our study as it may introduce bias. The study did not evaluate the impact of different interventions on functional outcomes, such as range of motion or strength. We recommend that ESPB be considered a preferred RA technique, given it demonstrated a higher incidence of success, enhanced intraoperative hemodynamic stability, superior postoperative analgesia, and diminished opioid requirements. Further research must explore the long-term outcomes and cost-effectiveness of these approaches. Future research should also consider assessing the efficacy of LS-ESPB in diverse surgical populations, including patients with different comorbidities and surgical procedures, as well as in ambulatory or fast-track surgical programs.

### 5.1. Conclusions

The LS-ESPB is a more effective analgesic approach than the PCB-SNB combination in unilateral LL surgeries. It has a higher incidence of success, more stable hemodynamics, better analgesia, elevated patient satisfaction, and comparable side effects.

## Data Availability

The dataset presented in the study is available on request from the corresponding author during submission or after publication.

## Clinical Trial Registration Code:

NCT06580704.

## References

[1] Lazar AE, Butiulca M, Farczadi L (2024). Challenges of the Regional Anesthetic Techniques in Intensive Care Units - A Narrative Review.. J Crit Care Med (Targu Mures)..

[2] Chaiwat O, Chanidnuan M, Pancharoen W, Vijitmala K, Danpornprasert P, Toadithep P (2019). Postoperative delirium in critically ill surgical patients: incidence, risk factors, and predictive scores.. BMC Anesthesiol..

[3] Doelakeh ES, Chandak A (2023). Risk Factors in Administering Spinal Anesthesia: A Comprehensive Review.. Cureus..

[4] Ribadiya R, Shah DD, Joshi DV, Parmar PD, Pancholi ND (2025). Efficacy and Safety of Combined Femoral (3-in-1) and Sciatic Nerve Block in Lower Limb Surgeries: A Clinical Study.. European Journal of Cardiovascular Medicine..

[5] Chen Y, Lin J, Chen X, Gong C, Xue F, Huang Y (2024). The addition of peripheral nerve blocks to routine spinal or general anesthesia was associated with decreased risks of major adverse events after total hip or knee arthroplasty: A retrospective, propensity score-matched cohort study.. Heliyon..

[6] Quan J, Yang S, Chen Y, Chen K, Yu S (2021). Ultrasound-Guided Comparison of Psoas Compartment Block and Supra-Inguinal Fascia Iliaca Compartment Block for Pain Management in Pediatric Developmental Dysplasia of Hip Surgeries.. Front Pediatr..

[7] Aytolign HA, Mersha AT, Ferede YA (2022). Analgesic efficacy of posterior and anterior psoas compartment block: Lumbar plexus versus three -in-one nerve block after lower limb orthopedic surgery under spinal anesthesia: A prospective cohort study.. Ann Med Surg (Lond)..

[8] Sun Q, Zhang C, Liu S, Lv H, Liu W, Pan Z (2023). Efficacy of erector spinae plane block for postoperative analgesia lumbar surgery: a systematic review and meta-analysis.. BMC Anesthesiol..

[9] Chin KJ, El-Boghdadly K (2021). Mechanisms of action of the erector spinae plane (ESP) block: a narrative review.. Can J Anaesth..

[10] Gupta A, Kaur J, Kumar R (2022). Unilateral sacral erector spinae plane block for lower limb surgery in children.. Anaesth Rep..

[11] Marrone F, Fusco P, Paventi S, Tomei M, Lolli S, Chironna E (2024). Combined lumbar and sacral erector spinae plane (LS-ESP) block for hip fracture pain and surgery.. Minerva Anestesiol..

[12] Aksu C, Gurkan Y (2019). Aksu approach for lumbar erector spinae plane block for pediatric surgeries.. J Clin Anesth..

[13] Chen Q, Beal EW, Okunrintemi V, Cerier E, Paredes A, Sun S (2019). The Association Between Patient Satisfaction and Patient-Reported Health Outcomes.. J Patient Exp..

[14] Yang JH, Sun Y, Yang YR, Qi LN, Li WY, Qin XZ (2024). The Analgesic Mechanism and Recent Clinical Application of Erector Spinae Plane Block: A Narrative Review.. J Pain Res..

[15] Abdella A, Arida E, Megahed NA, El-Amrawy WZ, Mohamed WMA (2022). Analgesia and spread of erector spinae plane block in breast cancer surgeries: a randomized controlled trial.. BMC Anesthesiol..

[16] Sorenstua M, Zantalis N, Raeder J, Vamnes JS, Leonardsen AL (2023). Spread of local anesthetics after erector spinae plane block: an MRI study in healthy volunteers.. Reg Anesth Pain Med..

[17] Karmakar MK, Reina MA, Sivakumar RK, Areeruk P, Pakpirom J, Sala-Blanch X (2021). Ultrasound-guided subparaneural popliteal sciatic nerve block: there is more to it than meets the eyes.. Reg Anesth Pain Med..

[18] de Leeuw MA, Slagt C, Hoeksema M, Zuurmond WW, Perez RS (2011). Hemodynamic changes during a combined psoas compartment-sciatic nerve block for elective orthopedic surgery.. Anesth Analg..

[19] Niyonkuru E, Iqbal MA, Zeng R, Zhang X, Ma P (2024). Nerve Blocks for Post-Surgical Pain Management: A Narrative Review of Current Research.. J Pain Res..

[20] Ozalp G, Tuncel G, Kaya M, Canoler O, Gulnerman G, Kadiogullari N (2004). The efficacy of psoas compartment block and extended femoral nerve sheath block for patient-controlled regional analgesia after total knee replacement.. Regional Anesthesia and Pain Medicine..

[21] Kukreja P, Deichmann P, Selph JP, Hebbard J, Kalagara H (2020). Sacral Erector Spinae Plane Block for Gender Reassignment Surgery.. Cureus..

[22] Kocum A, Turkoz A, Bozdogan N, Caliskan E, Eker EH, Arslan G (2010). Femoral and sciatic nerve block with 0.25% bupivacaine for surgical management of diabetic foot syndrome: an anesthetic technique for high-risk patients with diabetic nephropathy.. J Clin Anesth..

[23] Esmat I (2023). Ultrasound-Guided Techniques for Postoperative Analgesia in Patients Undergoing Laparoscopic Sleeve Gastrectomy: Erector Spinae Plane Block vs. Quadratus Lumborum Block.. Pain Physician Journal..

[24] Balakrishnan A, Chhabra A, Kumar A, Talawar P, Bhoi D, Garg H (2023). Comparison of continuous transmuscular quadratus lumborum block and continuous psoas compartment block for posterior total hip arthroplasty: A randomised controlled trial.. Indian J Anaesth..

[25] Cappelleri G, Cedrati VL, Fedele LL, Gemma M, Camici L, Loiero M (2016). Effects of the Intraneural and Subparaneural Ultrasound-Guided Popliteal Sciatic Nerve Block: A Prospective, Randomized, Double-Blind Clinical and Electrophysiological Comparison.. Reg Anesth Pain Med..

[26] Nagy A, Elshorbagy H, Hassanien A (2022). Efficacy of ultrasound guided erector spinae plane block on hemodynamic in patient undergoing abdominal surgery.. Minia Journal of Medical Research..

[27] Abotaleb AM, Negm EE, Abdelwahed WM (2023). A comparative study of preoperative ultrasound-guided lumbar erector spine plane block and preoperative ultrasound-guided caudal block for postoperative pain control in pediatric lower limb surgeries: A randomized controlled trial.. Egyptian Journal of Anaesthesia..

[28] Medhat MM, Kamel AAF, Salem DAE, Alagamy SA, Fathi HM (2023). The Analgesic Effects of Preemptive Ultrasound-Guided Pericapsular Nerve Group Block in Comparison with Erector Spinae Plane Block in Elderly Undergoing Hip Arthroplasty: A Randomized Controlled Trial.. Anesth Pain Med..

[29] Jin Y, Zhao S, Cai J, Blessing M, Sun Y, Hu S (2020). Efficacy of ultrasound-guided erector spinae plane block for perioperative pain control and short-term outcomes in lumbar laminoplasty.. medRxiv..

[30] Aksoy M, Dostbil A, Ince I, Ahiskalioglu A, Alici HA, Aydin A (2014). Continuous spinal anaesthesia versus ultrasound-guided combined psoas compartment-sciatic nerve block for hip replacement surgery in elderly high-risk patients: a prospective randomised study.. BMC Anesthesiol..

[31] Fu MY, Hao J, Ye LH, Jiang W, Lv YW, Shen JL (2023). Efficacy and Safety of Erector Spinae Plane Block for Perioperative Pain Management in Lumbar Spinal Surgery: A Systematic Review and Meta-Analysis of Randomized Controlled Trials.. J Pain Res..

[32] Zelenty WD, Li TY, Okano I, Hughes AP, Sama AA, Soffin EM (2023). Utility of Ultrasound-Guided Erector Spinae Plane Blocks for Postoperative Pain Management Following Thoracolumbar Spinal Fusion Surgery.. J Pain Res..

[33] Ganidagli S, Cengiz M, Baysal Z, Baktiroglu L, Sarban S (2005). The comparison of two lower extremity block techniques combined with sciatic block: 3-in-1 femoral block vs. psoas compartment block.. Int J Clin Pract..

[34] Canakci E, Unal D, Guzel Y (2017). The Effect of Unilateral Spinal Anaesthesia and Psoas Compartment with Sciatic Block on the Postoperative Pain Management in Total Knee Artroplastic Surgery.. Pain Res Manag..

[35] Ilfeld BM, Mariano ER, Madison SJ, Loland VJ, Sandhu NS, Suresh PJ (2011). Continuous femoral versus posterior lumbar plexus nerve blocks for analgesia after hip arthroplasty: a randomized, controlled study.. Anesth Analg..

[36] Marino J, Russo J, Kenny M, Herenstein R, Livote E, Chelly JE (2009). Continuous lumbar plexus block for postoperative pain control after total hip arthroplasty. A randomized controlled trial.. J Bone Joint Surg Am..

